# Reporting heterogeneity in self-assessed health among elderly Europeans

**DOI:** 10.1186/2191-1991-2-21

**Published:** 2012-10-05

**Authors:** Christian Pfarr, Andreas Schmid, Udo Schneider

**Affiliations:** 1 Department of Law and Economics, University Bayreuth, Chair of Public Finance, D-95440, Bayreuth, Germany; 2 WINEG – Scientific Institute of TK for Benefit and Efficiency in Health Care, D-22305, Hamburg, Germany

**Keywords:** Reporting heterogeneity, SHARE, Generalized ordered probit

## Abstract

**Introduction:**

Self-assessed health (SAH) is a frequently used measure of individuals’ health status. It is also prone to reporting heterogeneity. To control for reporting heterogeneity objective measures of true health need to be included in an analysis. The topic becomes even more complex for cross-country comparisons, as many key variables tend to vary strongly across countries, influenced by cultural and institutional differences. This study aims at exploring the key drivers for reporting heterogeneity in SAH in an international context. To this end, country specific effects are accounted for and the objective health measure is concretized, distinguishing effects of mental and physical health conditions.

**Methods:**

We use panel data from the SHARE-project which provides a rich dataset on the elderly European population. To obtain distinct indicators for physical and mental health conditions two indices are constructed. Finally, to identify potential reporting heterogeneity in SAH a generalized ordered probit model is estimated.

**Results:**

We find evidence that in addition to health behaviour, health care utilization, mental and physical health condition as well as country characteristics affect reporting behaviour. We conclude that observed and unobserved heterogeneity play an important role when analysing SAH and have to be taken into account.

## Background

Knowledge about the health status of individuals is paramount when health interventions are to be evaluated. Often, self-assessed health (SAH) is used as a key measure to this end. However, SAH is prone to inaccuracies due to reporting heterogeneity. Given an identical understanding of health-related questions and response style, self-assessed health would reflect (unobservable) true health which would make it a valid indicator. However, varying reporting behaviour leads to discrepancies between self-assessed health and the underlying true health. This may result in systematic differences in the stated health across population subgroups, even if the underlying true health status is identical. This gains importance when cross country comparisons are considered. The respective institutional or cultural setting can influence asymmetries between true and self-assessed health. Objective health measures as well as SAH show considerable differences between countries [1]. However, they do not reveal any sort of common pattern, which again directs the attention to potential causes for this finding.

This study investigates a wide range of potential causes for reporting heterogeneity in SAH. In detail, we focus on individual level socio-economic factors as well as on country level characteristics while controlling for objective measures of true health.

There are two aspects that are of special interest for the remainder of this article. The first relates to the relevance of reporting heterogeneity in SAH. The second elaborates on methodological issues that have to be considered when the extent and potential causes of this effect are to be captured econometrically.

In the literature, labour supply and retirement are typical fields in which the relevance of reporting heterogeneity is investigated. The main focus of these papers is on a possible endogeneity of health that may be driven by different valuations of individual health [2-4]. As it becomes clear from these studies, SAH is an invalid indicator, if current health and an objective measure are imperfectly correlated. Therefore, various studies try to obtain an objective measure of individual’s health stock [5]. Kerkhofs and Lindeboom [6] assume that endogeneity of health is driven by systematic misreporting in subjective health questions. Their results suggest that subjective health measures lead to biased estimates. In an extension of this work, Lindeboom and Kerkhofs [7] present evidence that the reporting of health problems is characterized by a great deal of heterogeneity and suggest to include more specific and therefore more objective health indicators. In a recent study, Ziebarth [8] provides evidence that compared to self-assessed health measures, concentration and thus heterogeneity in reporting health is significantly lower if other proxies of objective health, e.g. the SF12 or grip strength are used. Finally, Etile and Milcent [9] differentiate between the “production effect” of true health status and the effect of reporting heterogeneity. They show that the latter one is driven by individuals’ income.

With their study van Doorslaer and Jones [10] shift the focus towards methodological issues in the econometrics of reporting heterogeneity. They apply different estimation models to scale the responses of self-assessed health questions. Thereby the authors find that various sub-groups of the population systematically use different thresholds in classifying their health into a categorical measure. If population sub-groups use different reference points when answering health related questions this kind of heterogeneity may express itself either in a shift of the mean or in influencing the shape of the distribution [11]. The first effect is denoted as index shift and the distribution of the health measure shifts completely to the right or left, whereas the shape itself remains unchanged. The second effect is a cut-point shift, where reference points depend on the individual response behaviour and characteristics, which leads to a change in the shape of the distribution and thus to a non-parallel shift of cut-points. Several studies investigate the presence of such a cut-point or an index shift in the reporting of SAH. The results are quite mixed. While Lindeboom and van Doorslaer [11] find evidence for both kinds of shift depending on age and gender but not on income, education or language skills, Hernández-Quevedo et al. [12] only present evidence for the presence of an index shift. Bago d’Uva et al. [13,14] use anchoring vignettes to objectify health measures.^a^ Their results suggest that homogeneous reporting as well as a parallel shift of the reporting thresholds can be ruled out for all countries in the sample. Furthermore they conclude that when self-assessed health is used in the analysis of the distribution of doctor visits a bias seems to exist.

Our study investigates a wide range of potential causes for reporting heterogeneity in SAH while accounting for both cut-point and index shifts. In detail, we focus on individual level socio-economic factors as well as on country level characteristics while controlling for objective measures of true health.

Very similar to the aim of this study is the work by Schneider et al. [15]. They analyse how both socioeconomic factors and disease experiences influence the individual valuation of health. Applying a generalized ordered probit model to German panel data, they control for observed heterogeneity in the categorical health variable allowing the thresholds to depend on ex-ante identified explanatory variables. The results suggest strong evidence for cut-point shifts, especially regarding the experience with different kinds of illnesses. They also point to a gender specific perception and assessment of health.

One major finding of the presented studies is that self-reporting of health is affected by reporting heterogeneity. More specifically, the studies show differences between self-reported and the latent true health. The aim of this study is to have a closer look at the potential causes for these differences. To be able to investigate these differences a widely-used approach is the inclusion of more objective health measures as proxies for true health as proposed in the literature. Such objective measures can be based on illnesses diagnosed by a physician or other factors that are less susceptible to individual perceptions. Whereas Schneider et al. rely on a single index with a limited number of illnesses to capture true health we use separate and more comprehensive proxies for true mental and physical health, thereby covering multi-dimensional aspects of health and improving the quality of our objective health measure.

Furthermore, up to now all existing studies concerning cut-point and index shifts are based on data for single countries. Thus they are not able to control for the effects of cultural and institutional differences and whether heterogeneous reporting behaviour follows a common pattern.

Summarizing, our paper contributes to the existing literature that investigates the causes for reporting heterogeneity and cut-point as well as index shifts primarily in two ways; first, we provide improved objective health measures for physical and mental health. Second, by using the international SHARE panel data we have a closer look at country specific effects on reporting heterogeneity and include indicators such as out of pocket health expenditures. Furthermore, contrary to all but one study [15] we account for unobserved heterogeneity through panel data methods.

In the remainder of this paper, section two describes data and methods and gives first descriptive results on country differences. The results of estimating the driving factors of heterogeneity are presented and discussed in section 3 and the findings are summarized in a conclusion.

## Method

### Data description

In this study, we use data from the Survey of Health, Ageing and Retirement in Europe (SHARE)^b^. The full dataset contains information on more than 45,000 elderly Europeans (aged 50 years or older as well as spouses and partners irrespectively of their age) which was collected in two survey periods (2004/05 and 2006/07). A broad set of socioeconomics variables as well as in depth surveys of special topics make SHARE a valuable tool for research. In our case, health related questions are of particular interest. The survey embraces hard and soft health variables as well as psychological variables, information on health care utilisation and similar related topics. To mitigate the effects of item non-response we use the imputed version^c^ of this dataset [16].

For the analysis of reporting heterogeneity, we use the five-point categorical variable self-assessed health. This variable ranges from excellent (1) to poor (5). Using an unbalanced panel structure, we include socio-demographic characteristics, health related variables as well as country indicator variables as explanatory factors. The complete list of variables is presented in Table 1. The first group covers age and gender effects, the influence of education and income as well as family status and nationality. Possible nonlinearity in calendar age is captured by including a linear as well as a quadratic age term. To incorporate possible impacts of income, we refer to the relative income position of a household member based on the net household equivalent income [17]. The relative position depends on the median separately computed for each country and period. To compare education across countries, the International Standard Classification of Education (ISCED 1997) is used. The group of health-related variables consists of health behaviour, health condition and health care utilization. The variables for physical and mental conditions indicate multimorbidity and mental state of the respondent. Both are indices ranging from 0 to 100, with higher values indicating a worse condition (see chapter 2.2). Moreover, doctor visits and the number of nights in hospital are proxies for the utilization of health care. The reference categories represent no doctor visits or no night in hospital respectively. To account for cross-country variation not captured by the other variables, we include country fixed effects with France as reference. The other countries are Austria, Germany, Sweden, Netherlands, Spain, Italy, Denmark, Greece, Switzerland and Belgium. To control for differences in the health care systems, we incorporate the out-of-pocket health expenditures as well as the public health expenditures as percentage of total health expenditures in our regression. Finally, to avoid problems of endogeneity when considering the effects of retirement on SAH, we use the effective retirement age in each country as a macroeconomic indicator.^d^

**Table 1:** Variable description

**variable name**	**variable description**
SAH	Self-assessed health, 1 = excellent, 5 = poor
Survey Period	1 if survey period 2006/2007
Gender	1 if female
Age	Age in years
Age^2^	Age squared divided by 100
Marital status	1 if living with a partner or a spouse
Foreign	1 if foreign
Grandchildren	1 if respondent has got one or more grandchildren
Children	1 if respondent has got one or more children
Very low income	1 if income ≤ 50 % of the country’s median equivalent net household income
Low income	1 if income > 50 % but ≤ 75 % of the country’s median equivalent net household income
High income	1 if income > 125 % but ≤ 150 % of the country’s median equivalent net household income
Very high income	1 if income > 150 % of the country’s median equivalent net household income
Education1	1 if the level of education according to the ISCED scale is 3 or 4 (reference is ISCED category 1 and 2)
Education2	1 if the level of education according to the ISCED scale is 5 or 6 (reference is ISCED category 1 and 2)
Smoking	1 if respondent has ever been a daily smoker for at least one year
Drinking	1 if respondent has been drinking alcoholic beverages at least once or twice a week over the past six months
Physical activity	1 if respondent is engaged in vigorous physical activity like sports or heavy housework at least once a week
Physical condition	Index of respondents physical health status
Mental condition	Index of respondents mental health status
Doctor visits 1-3	1 if 1 to 3 doctor visits in the last 12 months
Doctor visits 4-11	1 if 4 to 11 doctor visits in the last 12 months
Doctor visits >11	1 if more than 11 doctor visits in the last 12 months
Hospital nights 1-6	1 if 1-6 nights in hospital in the last 12 months
Hospital nights 7-14	1 if 7-14 nights in hospital in the last 12 months
Hospital nights >14	1 if more than 14 nights in hospital in the last 12 months
Out-of-Pocket Exp.	Out-of-Pocket health expenditures as percentage of total expenditures on health
Public Health Exp.	Public health expenditures as percentage of total expenditures on health
Effective Retirement Age	Average effective age of retirement

The total number of observations from the two periods and eleven countries amounts to 53,931. As can be seen from Table 2, the mean of self-assessed health is 2.95, indicating a slight tendency to report a poor health status. Almost 50 % of the respondents state to have been a daily smoker for at least one year at some point in their life. Only 33 % report frequent drinking of alcoholic beverages during the past six months. Concerning health care utilization, 86 % visited a doctor at least once in the last twelve months, and 13 % had to stay in hospital for at least one night.

**Table 2:** Summary statistics

**N = 53,931**	***Mean***	***SD***
*Dependent variable*		
SAH	2.95	1.06
*Explanatory variables*		
Survey Period	0.49	0.50
Gender	0.56	0.50
Age	64.45	10.35
Age^2^	42.61	13.83
Marital status	0.76	0.43
Foreign	0.02	0.15
Grandchildren	0.63	0.48
Children	0.89	0.31
Very low income	0.15	0.35
Low income	0.18	0.38
High income	0.10	0.30
Very high income	0.28	0.45
Education1	0.31	0.46
Education2	0.19	0.39
Smoking	0.48	0.50
Drinking	0.33	0.47
Physical activity	0.50	0.50
Physical condition	49.87	9.91
Mental condition	49.93	9.95
Doctor visits 1-3	0.33	0.47
Doctor visits 4-11	0.36	0.48
Doctor visits >11	0.17	0.38
Hospital nights 1-6	0.07	0.25
Hospital nights 7-14	0.03	0.18
Hospital nights >14	0.03	0.16
Austria	0.06	0.23
Germany	0.10	0.30
Sweden	0.10	0.30
Netherlands	0.10	0.30
Spain	0.08	0.27
Italy	0.10	0.30
Denmark	0.08	0.27
Greece	0.11	0.31
Switzerland	0.04	0.20
Belgium	0.13	0.33
Out-of-Pocket Exp.	17.86	9.05
Public Health Exp.	71.98	6.76
Effective Retirement Age	60.89	1.96

### Computation of physical and mental condition indices

The identification of cut-point and index shift is only possible with an objective measure of true health. Therefore, we use a wide range of physical disabilities and mental states included in both waves of the SHARE dataset. Concerning the physical disabilities, we rely on questions regarding specific illnesses which were diagnosed by a physician. Our assessment of the individual’s mental condition is closely linked to emotional health or well-being which is captured through self-reported feelings and valuations of the personal life situation. The included aspects constitute core criteria for the EURO-D scale, a depression symptom scale, and the F32 code (depressive episode) of the ICD-10. For a detailed list of variables in use see Table 3 and Table 4.^e^

**Table 3:** Physical condition index

	** AUT**		** GER**		** SWE**		** NED**		** ESP**		** ITA**		** FRA**		** DEN**		** GRE**		** SUI**		** BEL**	
**Male**																						
heart attack	0.83	***	0.59	***	0.34	***	0.30	**	0.78	***	0.84	***	0.32	***	0.44	***	0.35	***	0.46	**	0.54	***
high blood pressure	−0.23	**	−0.22	***	−0.22	***	−0.31	***	−0.45	***	−0.45	***	−0.37	***	−0.37	***	−0.38	***	−0.53	***	−0.34	***
high blood cholesterol	−0.15		−0.18	*	−0.31	***	−0.13		−0.33	***	−0.25	***	−0.54	***	−0.46	***	−0.44	***	−0.31	**	−0.51	***
stroke	0.95	**	0.61	***	0.60	***	0.96	***	1.18	***	1.12	***	0.22		0.73	***	0.68	***	0.69	**	0.69	***
diabetes	0.54	***	0.08		−0.00		0.18		−0.14		0.11		0.07		0.19		−0.04		−0.28		0.27	**
chronic lung disease	1.51	***	0.51	***	0.51	**	0.77	***	0.64	***	0.58	***	0.62	***	0.51	***	0.36	*	0.94	***	0.61	***
asthma	0.41		0.33		0.08		0.37	*	−0.35		0.11		0.07		−0.10		−0.13		−0.06		0.31	
arthritis	0.49	**	0.78	***	0.53	***	0.94	***	0.44	***	0.10		0.32	***	0.35	***	0.16		−0.16		0.30	***
osteoporosis	0.78	**	0.40		0.18		0.97	***	0.17		0.63	**	0.01		1.28	**	0.08		0.51		0.12	
cancer	0.73	*	0.19		−0.16		−0.06		0.23		0.74	***	0.40	**	0.23		0.09		0.16		0.63	***
stomach/duodenal ulcer	0.83	**	0.26		0.10		−0.05		0.09		−0.29	*	0.06		0.09		−0.09		0.44		−0.13	
parkinson^+)^							1.05	*			0.99	**	1.00	*			1.27	**				
cataracts	−0.25		−0.02		0.03		−0.16		0.24		0.17		0.35	*	0.10		0.22		−0.01		−0.02	
hip fracture	0.18		0.28		0.54	**	1.08	*	0.61		−0.34		−0.08		0.19		0.43		0.25		0.59	*
other	0.31	**	0.55	***	0.12		0.48	***	0.29	***	0.24	**	0.28	***	0.06		0.22	*	0.12		0.42	***
***N***																						
**Female**																						
heart attack	0.48	**	0.31	**	0.22	**	0.34	**	0.61	***	0.80	***	0.61	***	0.67	***	0.77	***	0.33		0.93	***
high blood pressure	−0.13		−0.17	**	−0.21	***	−0.13	*	−0.38	***	−0.17	***	−0.19	***	−0.38	***	−0.28	***	−0.29	***	−0.41	***
high blood cholesterol	−0.02		−0.31	***	−0.31	***	−0.11		−0.28	***	−0.31	***	−0.30	***	−0.30	***	−0.28	***	−0.51	***	−0.37	***
stroke	0.77	*	0.56	**	0.47	**	0.62	**	0.59	*	1.21	***	0.18		0.94	***	0.90	***	0.53		0.50	*
diabetes	0.78	***	0.55	***	0.10		0.18		0.29	**	0.40	***	0.12		0.07		−0.14		−0.10		0.16	
chronic lung disease	0.63	**	0.39	**	1.14	***	0.66	***	0.38	*	0.49	***	0.38	**	0.53	***	0.45	**	0.07		0.57	***
asthma	0.69	**	0.37	*	0.14		0.59	***	0.11		−0.03		−0.13		0.04		0.09		0.02		0.13	
arthritis	0.66	***	0.72	***	0.42	***	0.81	***	0.48	***	0.21	***	0.21	***	0.28	***	0.20	***	0.10		0.53	***
osteoporosis	0.22	*	0.74	***	0.10		0.34	***	0.22	*	0.26	***	−0.04		0.21		−0.15	**	0.35	*	0.08	
cancer	0.74	*	0.52	***	−0.03		0.31	*	0.76	**	0.44	**	0.19		−0.07		0.13		0.08		0.73	***
stomach/duodenal ulcer	0.78	**	0.30		0.21		0.41		0.21		−0.09		0.63	***	0.18		−0.01		0.23		0.04	
parkinson^+)^									0.99		0.82	*	0.69		0.93	**	1.33	**	0.54		1.35	***
cataracts	−0.10		−0.02		0.18	*	0.22		0.29	*	0.45	***	0.29	**	0.29	**	0.30	**	−0.10		0.20	
hip fracture	1.40	***	0.75		0.19		−0.15		0.78	***	0.30		0.60	**	0.22		0.20		0.66		1.18	***
other	0.52	***	0.50	***	0.36	***	0.45	***	0.25	***	0.19	**	0.09		0.01		−0.03		0.21	**	0.21	**
**N**	785		1372		1470		1432		1212		1629		1660		1436		1822		806		1730	

^+)^ Variable dropped for some countries due to collinearity.

**Table 4:** Mental condition index

**Male**	** AUT**		** GER**		** SWE**		** NED**		** ESP**		** ITA**		** FRA**		** DEN**		** GRE**		** SUI**		** BEL**	
sad or depressed last month	0.06		0.18	*	0.03		0.25	**	0.19		0.32	***	0.10		0.13		0.34	***	0.24		0.04	
felt would rather be dead	0.61		−0.06		0.54	**	0.25		0.66	**	0.37	**	0.41	***	0.73	**	0.58	*	0.17		0.35	**
feels guilty	0.62	**	0.04		−0.05		0.18		−0.39	**	−0.05		−0.08		0.08		−0.07		−0.07		−0.06	
trouble sleeping	0.66	***	0.45	***	0.39	***	0.37	***	0.28	**	0.31	***	0.25	***	0.21	**	0.26	**	0.32	**	0.28	***
less or same interest in things	0.29		0.39	**	0.49	***	0.16		0.12		0.14		0.01		0.05		0.32	***	−0.08		0.25	*
irritability	−0.01		0.15		0.01		−0.06		0.24	**	−0.00		−0.14		0.04		−0.09		−0.25	*	0.06	
no appetite	−0.50		−0.27		−0.61	***	−0.85	***	−0.32	**	−0.32	**	−0.46	***	−0.42	**	−0.28		−0.82	***	−0.48	***
fatigue	0.78	***	0.55	***	0.58	***	0.73	***	0.31	***	0.62	***	0.70	***	0.54	***	0.30	***	0.53	***	0.94	***
difficulties concentrating																						
on entertainment	0.09		−0.15		0.27	*	0.33	**	0.19		0.12		0.25	*	0.28		−0.04		0.39	**	0.03	
on reading	0.59	**	0.23		0.11		0.11		0.50	***	0.35	***	0.12		0.41	***	0.32	**	0.17		0.38	***
no enjoyment	−0.04		0.25	**	−0.07		0.21		0.12		0.13		0.18		0.28	**	0.16		0.27		−0.06	
tearfulness	−0.07		0.17		−0.05		0.08		0.07		−0.13		−0.06		0.12		−0.29	*	0.22		0.07	
***N***																						
**Female**																						
sad or depressed last month	0.46	***	0.17	**	0.11		−0.03		0.16	*	0.23	***	0.01		0.24	***	0.27	***	0.07		0.01	
felt would rather be dead	0.32		0.33		0.22		0.28		0.16		0.64	***	0.23	**	0.43	**	0.14		0.27		0.39	***
feels guilty	−0.01		−0.06		−0.07		−0.07		−0.06		−0.09		−0.14	*	−0.06		−0.15		−0.17		−0.08	
trouble sleeping	0.48	***	0.30	***	0.26	***	0.39	***	0.49	***	0.20	***	0.28	***	0.24	***	0.33	***	0.26	**	0.25	***
less or same interest in things	0.23		−0.08		0.32	**	0.01		0.21	*	0.10		0.08		0.45	***	−0.01		0.26		0.07	
irritability	−0.13		−0.13		−0.02		0.21	*	−0.04		−0.24	***	−0.17	**	0.02		−0.34	***	−0.11		−0.08	
no appetite	0.12		−0.39	***	−0.36	**	−0.32	**	−0.30	**	−0.02		−0.35	***	−0.32	**	−0.44	***	−0.66	***	−0.17	
fatigue	0.69	***	0.72	***	0.63	***	0.74	***	0.32	***	0.67	***	0.73	***	0.43	***	0.37	***	0.54	***	0.68	***
difficulties concentrating																						
on entertainment	−0.06		0.01		−0.19		0.44	***	0.27	**	0.14		0.24	**	0.13		0.39	***	−0.26		0.13	
on reading	0.47	**	0.46	***	0.42	***	0.04		0.16		0.33	***	0.17	*	0.45	***	0.30	***	0.56	***	0.32	***
no enjoyment	0.17		0.09		0.12		−0.00		0.37	***	0.10		0.18		0.29	*	0.16		0.62	***	0.23	**
tearfulness	−0.25	**	0.19	**	0.08		0.06		0.14		0.15	*	0.08		0.08		0.11		−0.14		0.07	
**N**	785		1359		1416		1419		1153		1597		1578		1407		1771		799		1704	

* *p* < 0.1, ** *p* < 0.05, *** *p* < 0.01.

The procedure applied is based on the work of Kerkhofs and Lindeboom [6] and Jürges [1]. We expand their approach by constructing two separate indices – one for physical and one for mental conditions – to objectify the reporting of illnesses or emotional distress. In a first step, we regress the binary indicator “limited activities” separately on the sets of physical and mental variables.^f^ The regressions for the physical and mental conditions index are run separately by country, gender and survey period, using standard probit models. By doing so, we account for different prevalence rates of specific physical and mental conditions, gender differences and time effects. The results of the index regression for the period 2006/2007 are presented in Table 3 and Table 4.

The results are reported separately for males and females and for all countries. As one can see, there is large variation between the countries. For both indices, we find gender differences regarding the magnitude, the sign and the significance of the coefficients. For males, the magnitude of the heart attack coefficient in the physical index regression ranges from 0.84 in Italy to 0.30 in the Netherlands. The highest impact for stroke is found in Spain (1.18), while for France we find no significance at all. Some forms of diseases only show an impact in a few countries, e.g. hip fracture, stomach ulcer or cancer. For women, osteoporosis reveals changing signs. While the influence is highly significant and positive (0.74) for German women, it is negative for Greece (-0.15). Considering the mental condition index, a similar pattern is found for men and the attitude “feels guilty”. While Austrians are affected negatively the picture is reverse for Spain. Further items like difficulties to concentrate on entertainment, no enjoyment and tearfulness are only partly significant.

In a second step, the coefficients of the respective sub-regressions are used to predict a “latent” variable of the true health status for each individual. The predicted values are transformed by using an inverse log transformation resulting in positive values. We compute the final indices by combining the results of the country sub-regressions, i.e. we standardize the results across countries, but separately for gender and year. The final physical and mental indices range from 0 to 100 with mean 50 and a standard deviation of 10 if all countries are considered. Country-specific means can deviate from this value. A higher index value indicates a higher degree of multimorbidity or poor mental state respectively.

### Cross-country comparison

**Figure 1:**
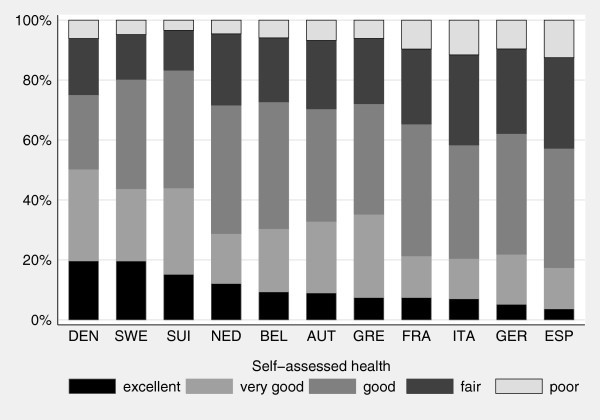
Distribution of self-assessed health by country.

For the further analysis of reporting heterogeneity across European countries, it is important to take a closer look at the distribution of self-assessed health. To make a cross-country comparison meaningful, we compute age-gender-standardized distributions of SAH. Figure 1 shows the standardized distribution of SAH across countries pooled for both observation periods.

Following the presented picture, the healthiest individuals live in Denmark and Sweden. This is in line with the results presented in Jürges [1]. It is obvious that there exists large variation across the countries. While a fraction of 50 % of the Danish population reports very good or better health, the proportion drops below 20 % for Spain. On the contrary, only about 18 % of the Swiss state their health as fair or poor whereas the least healthy population seems to be in Italy and Spain (more than 40 % reporting a health status below good).

**Figure 2:**
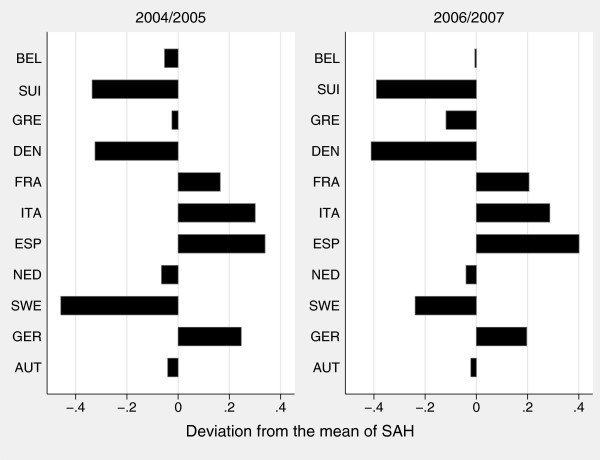
Deviation from the mean of self-assessed health by country.

If reported differences are not only related to differences in true health, they are likely to depend also on variations in the interpretation of the categories. Therefore, we aim at identifying factors responsible for these differences in the evaluation of self-assessed health across countries. While Figure 1 only shows the distribution of self-assessed health categories across European countries, Figure 2 represents the deviation from the age-gender standardized mean of SAH.

Here, the differences between the countries are distinctly visible. The countries rating their health lower than average are France, Germany, Italy and Spain. In the period 2004/2005, Sweden shows the largest negative deviation from the mean. This indicates that based on a self-reported measure Sweden has the healthiest population on average, even healthier than Denmark. The picture changes, however, when the period of 2006/2007 is considered. Here, the magnitude of the deviation for Sweden has come down to a half, a fact not visible from the pooled presentation in Figure 1. Between the observation periods, the devations are stable for Belgium, the Netherlands and Austria.

**Figure 3:**
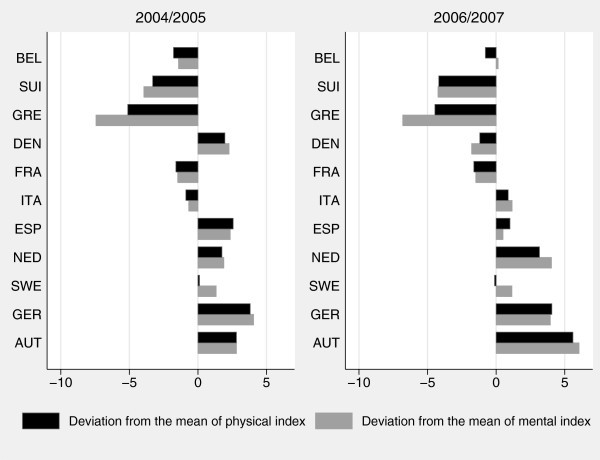
Deviation from the mean of mental and physical health index by country.

With respect to objective health measures, the country deviations from the standardized mean of 50 for our physical respectively mental condition indices are presented in Figure 3. Obviously, there exist large differences compared to the SAH figure. For the period 2004/2005, in Sweden and Denmark, the countries with the best self-assessed health, the picture for the objective health indices is completely different. According to this, reported health in those countries is overrated compared to the underlying true health. A similar picture results for Austria while for France and Italy the interpretation is that reported health underrates true health. For the period 2006/2007, the results change slightly. However, some countries change from a negative to a positive deviation and vice versa. Moreover, according to Figure 3, true health has significantly declined in Austria and the Netherlands.

Finally, for most of the countries, we observe a higher variation for the mental condition index. This may be due to the fact that the physical index is based on illnesses diagnosed by a physician, whereas the mental index builds on self-reported criteria, which are less strictly defined and as such much more prone to cultural influences.

### Estimation approach

One obstacle to the traditional ordered probit model used to analyse categorical variables is the single index or parallel lines assumption [18]. The coefficient vector is assumed to be the same for all categories of the dependent variable. In detail, this can be interpreted as a shift in the cumulated distribution function through an increase of an independent variable, i.e. the distribution shifts to the right or left, but there is no shift in the slope. By relaxing this assumption and allowing the indices to differ across the outcomes one gets the generalized ordered probit model [19].^g^

In our case, let *y* be the ordered categorical outcome of SAH, *y* ∈ {1, 2,…, *J*}. *J* denotes the number of distinct categories. Underlying the observed variable *y* is the latent health status of the respondent y^*^. While we use panel data, we apply a random effects generalized ordered probit model. For the data at hand, *i* denotes the cross-sectional unit and *t* the time dimension:

(1) $$\begin{array}{l} y_{\mathrm{it}}^{*}={x^{\prime }}_{\mathrm{it}}\beta +\varepsilon_{\mathrm{it}}\\ \varepsilon_{\mathrm{it}}=u_{\mathrm{it}}+\alpha_{i}\\ y_{\mathrm{it}}=j\Leftrightarrow {\tilde{\kappa }}_{j-1}+{x^{\prime }}_{\mathrm{it}}\gamma_{j-1}\leq y_{\mathrm{it}}^{*}\leq {\tilde{\kappa }}_{j}+{x^{\prime }}_{\mathrm{it}}\gamma_{j},j=1,\ldots ,5\\ E\left[\varepsilon_{\mathrm{it}}\right]=0\\ Var\left[\varepsilon_{\mathrm{it}}\right]=1+\sigma_{\alpha }^{2}\\ Corr\left[\varepsilon_{\mathrm{it}};\varepsilon_{\mathrm{is}}\right]=\rho =\frac{\sigma_{\alpha }^{2}}{1+\sigma_{\alpha }^{2}} \end{array}$$

The βs are the unknown coefficients. While in the traditional ordered probit model the unknown threshold parameters are constant, the threshold parameters in the generalized model к_*ij*_ are individual specific and depend on the covariates:^h^

(2) $$\kappa_{\mathrm{ij}}={\tilde{\kappa }}_{j}+{x^{\prime }}_{\mathrm{it}}\gamma_{j}\text{,}$$

Here, γ_j_ are the influence parameters of the covariates on the thresholds and ${\tilde{\kappa }}_{j}$ represents a constant term. It is important to note that the coefficients of the covariates and the threshold coefficients cannot be identified separately if the same set of variables *x* is used.

(3) $$\begin{array}{l} y_{\mathrm{it}}=j\Leftrightarrow {\tilde{\kappa }}_{j-1}+{x^{\prime }}_{\mathrm{it}}\gamma_{j-1}\leq y_{\mathrm{it}}^{*}={x^{\prime }}_{\mathrm{it}}\beta +\varepsilon_{\mathrm{it}}\leq {\tilde{\kappa }}_{j}+{x^{\prime }}_{\mathrm{it}}\gamma_{j},\\ \text{with}\;j=1,\ldots ,5,\;t=1,\ldots ,T,\;i=1,\ldots ,N\text{.} \end{array}$$

From this, it is clear that β_j_ = β – γ_j_. Following Williams [20], this results in the estimation of *J*-1 binary probit models (see section 4). For our purpose, this method enables us to control for individual heterogeneity in the β-parameters and hence for heterogeneity across the categories of the dependent variable. Consequently, the advantage of using panel data in combination with a generalization of the ordered probit model is to distinguish between two kinds of heterogeneity. First, unobserved individual heterogeneity is captured by our random effects specification. Second, varying cut-points and beta coefficients characterize the observed heterogeneity in the reporting of self-assessed health.

Individual specific β coefficients imply a cut-point shift if the relative position of these thresholds changes. If we find a parallel shift in the thresholds instead, the distribution of SAH shifts completely to the left or the right (index shift). The distinction between both kinds of shifts is of high relevance if the parallel shift cannot be separated from changes in the relative position of the thresholds [11]. To identify cut-point and index shifts, Lindeboom and van Doorslear [11] assume that true health is conditioned by objective health measures. In our generalized model, we first test for a cut-point shift related to our mental and physical health index. If the hypothesis of a cut-point shift is rejected, an index shift exists.

The iterative procedure to identify variables that drive the heterogeneity was first proposed by Williams [20] for cross-section data. In an extension, Pfarr et al. [21] combine this with the random-effects specification of the generalized ordered probit model by Boes [19].^i^

## Empirical evidence

### Results

Table 5 presents the results of the estimation of a generalized ordered probit model for panel data. In the table, we display the results of the four underlying binary models. The first model estimates category 1 (excellent) versus categories 2,., 5, the second model categories 1 and 2 (excellent and very good) versus 3,., 5 and so on. The interpretation of a negative coefficient for the model 1-2 versus 3-5 is as follows: the negative value indicates a higher probability to report categories 1 or 2, while a positive coefficient indicates a higher probability of reporting the worse health status.

**Table 5:** Estimation results of the generalized ordered probit model

**SAH**	**1 vs. 2-5**		**1-2 vs. 3-5**		**1-3 vs. 4-5**		**1-4 vs. 5**	
	**Coeff.**	***p* value**	**Coeff.**	***p* value**	**Coeff.**	***p* value**	**Coeff.**	***p* value**
Survey Period	0.037	(0.155)	0.060	(0.003)	0.228	(0.000)	0.158	(0.000)
**Gender**	0.088	(0.000)	0.088	(0.000)	0.088	(0.000)	0.088	(0.000)
Age	0.079	(0.000)	0.084	(0.000)	0.061	(0.000)	0.016	(0.276)
Age^2^	−0.047	(0.000)	−0.048	(0.000)	−0.035	(0.000)	−0.006	(0.561)
**Marital status**	0.056	(0.001)	0.056	(0.001)	0.056	(0.001)	0.056	(0.001)
Foreign	−0.027	(0.707)	0.145	(0.014)	0.190	(0.002)	0.321	(0.000)
Grandchildren	0.028	(0.279)	0.036	(0.080)	0.097	(0.000)	−0.032	(0.331)
**Children**	−0.017	(0.468)	−0.017	(0.468)	−0.017	(0.468)	−0.017	(0.468)
**Very low income**	0.106	(0.000)	0.106	(0.000)	0.106	(0.000)	0.106	(0.000)
**Low income**	0.090	(0.000)	0.090	(0.000)	0.090	(0.000)	0.090	(0.000)
**High income**	−0.053	(0.057)	−0.053	(0.057)	−0.053	(0.057)	−0.053	(0.057)
**Very high income**	−0.132	(0.000)	−0.132	(0.000)	−0.132	(0.000)	−0.132	(0.000)
**Education1**	−0.230	(0.000)	−0.230	(0.000)	−0.230	(0.000)	−0.230	(0.000)
Edcuation2	−0.411	(0.000)	−0.508	(0.000)	−0.492	(0.000)	−0.321	(0.000)
Smoking	0.046	(0.040)	0.084	(0.000)	0.078	(0.000)	0.165	(0.000)
**Drinking**	−0.116	(0.000)	−0.116	(0.000)	−0.116	(0.000)	−0.116	(0.000)
Physical activity	−0.308	(0.000)	−0.356	(0.000)	−0.447	(0.000)	−0.548	(0.000)
Physical health index	0.016	(0.000)	0.023	(0.000)	0.034	(0.000)	0.032	(0.000)
Mental health index	0.033	(0.000)	0.042	(0.000)	0.051	(0.000)	0.052	(0.000)
Doctor visits 1-3	0.366	(0.000)	0.280	(0.000)	0.177	(0.000)	−0.074	(0.222)
Doctor visits 4-11	0.831	(0.000)	0.778	(0.000)	0.719	(0.000)	0.384	(0.000)
Doctor visits >11	1.045	(0.000)	1.107	(0.000)	1.174	(0.000)	0.808	(0.000)
**Hospital nights 1-6**	0.188	(0.000)	0.188	(0.000)	0.188	(0.000)	0.188	(0.000)
**Hospital nights 7-14**	0.322	(0.000)	0.322	(0.000)	0.322	(0.000)	0.322	(0.000)
**Hospital nights >14**	0.581	(0.000)	0.581	(0.000)	0.581	(0.000)	0.581	(0.000)
Austria	−0.437	(0.037)	−0.832	(0.000)	−1.913	(0.000)	−1.511	(0.000)
Germany	0.064	(0.622)	−0.281	(0.003)	−1.069	(0.000)	−1.002	(0.000)
Sweden	−0.975	(0.000)	−1.087	(0.000)	−2.175	(0.000)	−1.330	(0.000)
Netherlands	−0.437	(0.000)	−0.432	(0.000)	−0.407	(0.000)	−0.848	(0.000)
Spain	0.023	(0.945)	−0.207	(0.403)	−2.336	(0.000)	−1.485	(0.001)
Italy	−0.345	(0.271)	−0.241	(0.307)	−1.969	(0.000)	−1.242	(0.002)
Denmark	−0.850	(0.000)	−1.213	(0.000)	−1.616	(0.000)	−1.085	(0.000)
Greece	−0.199	(0.763)	−0.581	(0.238)	−4.329	(0.000)	−2.378	(0.006)
Switzerland	−0.786	(0.134)	−0.962	(0.014)	−4.289	(0.000)	−2.576	(0.000)
Belgium	−0.371	(0.160)	−0.521	(0.009)	−2.066	(0.000)	−1.431	(0.000)
Out-of-Pocket Exp.	0.015	(0.493)	0.007	(0.667)	0.143	(0.000)	0.074	(0.010)
**Public Health Exp.**	−0.002	(0.522)	−0.002	(0.522)	−0.002	(0.522)	−0.002	(0.522)
Effective Retirement Age	0.020	(0.198)	0.029	(0.027)	0.063	(0.000)	0.070	(0.001)
_cons	−4.957	(0.000)	−7.326	(0.000)	−12.254	(0.000)	−11.791	(0.000)
ρ	0.417	(0.000)						
*N*	53931							

Note: For those variables printed in bold the parallel lines assumption holds.

According to our iterative procedure, we end up with 13 variables to be constrained in the estimation. This means that these variables are assumed to have equal effects across the categories of self-assessed health and hence across the four binary models. In detail, the parallel lines assumption holds for *Gender*, *Marital status*, *Children*, *Education1*, all variables of relative income, *Drinking* and the three variables covering hospital nights. In addition, public health expenditures is the only country specific indicator that meets the parallel lines assumption. However, it is not significant.

Regarding the income effects, individuals from households with an income lower than 75 % of the median tend to report a poorer health status compared to the reference category (income > 75 % but ≤ 125 % of the country’s median equivalent net household income). For households with a higher income (more than 125 % of median), we find a significantly negative impact. The interpretation is that ceteris paribus individuals from households with high income tend to report a better health status. Taking the income-health nexus into account, this result is not surprising. The variable reflecting moderate as well as frequent consumption of alcoholic beverages indicates a tendency to report a better health status.

Variables for which the parallel lines assumption is not imposed drive the observed heterogeneity in self-assessed health. The effects of these variables are allowed to vary across the four binary regressions, meaning that the coefficients may differ with respect to magnitude, sign and level of significance. Within the group of socioeconomic variables *Education2*, *Smoking* and *Physical activity* show varying influence on the distinct categories of SAH. For the first variable – *Education2* – the effect is significantly negative across all equations. The magnitude of the corresponding coefficients differs only slightly. Higher education – in terms of a university degree or vocational training – thus leads to a better self-reported health status. The signs of the other two factors – *Smoking* and *Physical activity* – are as expected. We find positive coefficients for (current or past) smokers and negative ones for physical activities. The magnitude for both variables increases in absolute terms and is highest for equation 1-4 vs. 5. Hence, poor health is reported more often by smokers, but less often for individuals doing sports or heavy housework. Related to the age structure of the SHARE dataset, the effect of smoking shows the long-lasting impact of adverse health behaviour.

Health care utilization of outpatient care shows large and significant effects. While 1-3 doctor visits in the last 12 months are only significant for the first three equations, more than 4 visits are significant for all regressions. Comparing 1-3 with 4-11 visits, the coefficients of the latter factor are more than twice as high. In addition, the effect is stronger for individuals visiting a doctor more than once a month on average. Using a sample of elderly Europeans, these effects are not surprising and correspond to an increasing morbidity at higher age.^j^

Both health indices are highly significant and positive over all equations. It is obvious that the coefficients for the mental condition index are always higher than the ones for the physical condition index. Individuals suffering from mental disorders hence may report to be more limited with respect to their health than individuals with diagnosed physical diseases. Thus, in particular mental effects drive the reporting heterogeneity. Concerning cut-point and index shifts, both indices enable us to incorporate proxies of true health. As both proxies are varying across the categories, we are able to rule out the possibility of a parallel shift in the thresholds (index shift). Hence, comparing answers on self-assessed health with illness related as well as mental health related questions gives evidence for the hypothesis that heterogeneity is driven by objective health measures.

We also include 10 country dummy variables with France as reference category. This enables us to control for cultural characteristics as well as to take peculiarities of the health care systems into account. Those countries with the healthiest population (Denmark, Sweden and Switzerland) show a distinct pattern, namely negative and highly significant coefficients for all four equations compared to France. Individuals in those countries are more likely to report a better health status. The influence is highest when deciding between health categories excellent and very good on the one hand, versus good to poor on the other hand. Taking into account Figure 1, this resembles the fact that over 40 % of the people in these countries state to be in the two best health categories. Opposite to these findings, we obtain alternating signs of the coefficients for some countries. For example, in relation to France, Germany tends to report excellent status less often, while the remaining coefficients show a trend towards reporting the middle category. This comes along with the highest negative impact for the last equation, meaning that Germans state poor health less likely than the French. In relation to the reference country, Germans neither report excellent nor poor health status very likely. The findings for Greece are somewhat different, because a positive coefficient for the first equation is followed by a negative for the second, while the last two are positive again. This would imply that Greeks prefer to state very good instead of excellent health, but are less likely to classify themselves into the middle category.

Regarding the two variables that cover differences in health systems, only the out-of-pocket expenditures show a varying and partly significant effect. The level of private out-of-pocket health expenditures is relevant when differentiating between the middle category and very poor health. This implies that the higher the out-of-pocket expenditures the worse the individual reported health status. In the contrary, public health expenditures do not seem to be of relevance for the individual perception of health.

Finally, also the effective retirement age effects self-reported health. The higher the effective retirement age, the higher is the likelihood to state a lower health status. This is plausible, considering the evidence presented by Coe and Zamarro [4]. They find that retired people have a tendency to report a better health status. In our data, the sample from a country with a higher effective retirement age is likely to embrace a larger share of still working elderly individuals who tend to report a worse health status.^k^ The influence of unobserved heterogeneity is confirmed by the high significance of the correlation of the error terms ρ.

## Discussion

The results presented above have to be critically assessed considering potential limitations that are caused by the chosen method and the underlying data. Regarding the construction of the two objective health measures the dependent variable “limited activities” has to be briefly discussed. In its ideal form this dependent variable should be robust to country specific response styles. As Table 6 indicates there is some variation regarding the prevalence of “limited activities” between countries and across gender and over time. The question is whether such a variation might be partly due to factors such as country specific response styles.

**Table 6:** Country means of “limited activities”

**Country**	**Period 1: Means**		**Period 2: Means**	
	**Male**	**Female**	**Male**	**Female**
Austria	0.427	0.500	0.498	0.536
Germany	0.456	0.518	0.465	0.491
Sweden	0.408	0.465	0.402	0.446
Netherlands	0.393	0.495	0.452	0.501
Spain	0.423	0.497	0.379	0.459
Italy	0.345	0.439	0.375	0.462
France	0.366	0.400	0.358	0.385
Denmark	0.425	0.481	0.345	0.386
Greece	0.251	0.317	0.250	0.296
Switzerland	0.311	0.361	0.289	0.331
Belgium	0.355	0.406	0.379	0.421
Overall	0.379	0.443	0.377	0.424

Summarizing Table 6, there is no clear picture regarding country specific response styles. Following this and considering the variables at hand we are confident that this is the best measure available to serve as a proxy for health status. The lack of a perfect dependent variable is common to all related studies.

In line with Jürges [1], we assume that self-reported diagnoses reflect true health status. Under this assumption, the resulting health indices are robust to differences in diagnosing illnesses across countries as they are constructed seperately for each country.

There may be concerns that the self-reported symptoms which form the basis for the mental health index might be less reliable than diagnosed diseases. However, the symptoms used to construct the mental condition index are core elements of psycological classification systems such as the EURO-D or also the F32 code of the ICD-10, the international statistical classification of diseases and realted health problems. This strongly supports our assumption that the self-reported diagnoses and the self-reported symptoms respectively are reliable measures of true health.

As a last issue concerning the health indices time effects have to be taken into account. That is, patients may get used to certain conditions and at the same time change their attitude towards a certain health status. Unfortunately, the dataset does not capture any information on the time elapsed since the condition has been diagnosed for the first time. The same applies to the mental symptoms. We tried to capture at least the information available from the two period panel data by constructing separat indices for each survey year.

With respect to the estimation endogeneity problems regarding individuals’ health care use and health behaviour might exist. Therefore, we estimated three alternative specifications of the presented model controlling for these issues. Specification I included neither variables on health care utilization nor variables on health behaviour. Specification II and III excludes either health care utilization or health behaviour respectively. The results show no systematic differences between the coefficients of the physical and mental condition indices, of the income variables, of age and of country fixed effects as well as of country specific macro indicators.^l^ The findings support the assumption that potential endogeneity does not affect the presented results in the results section.

Regarding the international perspective of the study, only few country level indicators are available for the full set of countries and for both survey periods, they may capture unobserved country effects, too. Furthermore, a larger number of countries would help to reduce the remaining uncertainty. To check the robustness of our results, we also estimated a model without the macro level indicators, including only country dummies. When including the three macro level indicators only the coefficients for the country dummies become less significant and varied in their magnitude. This is especially the case for countries such as Spain and Greece, which exhibit an exceptional high proportion of out-of-pocket expenditures. This means that health systems specific variables capture a considerable portion of the effect which is otherwise summarized by the country dummies.

## Conclusions

Evaluation of health interventions is often based on variables such as self-assessed health (SAH). However, SAH is prone to inaccuracies due to reporting heterogeneity which may result in differences of the stated health across population subgroups, even if the underlying true health status is identical. As the elderly typically face the highest level of morbidity and have usually a long history of dealing with their health issues, reporting heterogeneity is a very likely problem in this group. Moreover, it seems of high interest to see how institutional and cultural settings influence the divergence of true and self-assessed health. To account for such differences we conduct a comparison across eleven European countries using the Survey of Health, Ageing and Retirement in Europe (SHARE) for a panel analysis. We estimate a generalized ordered probit model to identify potential cut-point shifts in the health distribution. To account for true health, we compute indices for mental and physical conditions and include these together with measures for health care utilization, socio-demographic variables and country fixed effects to evaluate their relevance for reporting heterogeneity. While observed heterogeneity is reflected in the cut-point shifts, we are able to account for unobserved heterogeneity by using a random effects specification.

The results of the generalized ordered probit model indicate that cut-point shifts are present in the reporting of self-assessed health across countries. For example, in Germany individuals systematically report a lower health status, whereas Dutch respondents show a higher probability to opt for the best category. For both health indices, we find evidence of reporting heterogeneity. This means that a worse objective health status not only leads to a lower perception of own health but also that the impact of the effect varies between the categories of SAH. Moreover, the magnitude of mental health problems exceeds the effect of the pure physical health index. We find further evidence for reporting heterogeneity when looking at aspects like health care utilization and health relevant behaviour. Hence, our results support the view that there exists a gap between true and reported health.

As country effects may still reflect differences in health systems as well as unaccounted cultural variation the next step will be to elaborate on these two aspects in more detail. This would allow deriving policy implications focusing on differences in health care systems from an international perspective.

## Endnotes

^a^Here, respondents are asked to rate hypothetical descriptions of a fixed level of a latent construct [22,23].

^b^This paper uses data from SHARELIFE release 1, as of November 24th 2010 or SHARE release 2.5.0, as of May 24th 2011. The SHARE data collection has been primarily funded by the European Commission through the 5th framework programme (project QLK6-CT-2001- 00360 in the thematic programme Quality of Life), through the 6th framework programme (projects SHARE-I3, RII-CT- 2006-062193, COMPARE, CIT5-CT-2005-028857, and SHARELIFE, CIT4-CT-2006-028812) and through the 7th framework programme (SHARE-PREP, 211909 and SHARE-LEAP, 227822). Additional funding from the U.S. National Institute on Aging (U01 AG09740-13S2, P01 AG005842, P01 AG08291, P30 AG12815, Y1-AG-4553-01 and OGHA 04-064, IAG BSR06-11, R21 AG025169) as well as from various national sources is gratefully acknowledged (see www.share-project.org for a full list of funding institutions).

^c^A missing value is imputed five times, resulting in five complete datasets including all imputed and not-imputed variables [24]. The procedure is based on the fully conditional specification method (FCS) of van Buuren et al. [25].

^d^For each observation period the out-of-pocket as well as the public health expenditures were taken from the World Development Indicators Database (WDI). The effective retirement age as weighted average for each year is provided by Keese [26].

^e^In contrast to Jürges [1], we refrain from using the variables walking speed and grip strength. These variables show a large number of missing values (about 10 % for grip strength) or are not available for respondents younger than 75. Jürges assumes that all individuals for which walking speed is not measured to have a normal walking speed. Further, the BMI is not included in our specification because first, it may influence both, mental and physical conditions. Second, the BMI can be seen as the result of individual behaviour rather than a diagnosed disease. Moreover, especially obesity is closely related to diseases such as diabetes, cholesterol, arthritis or heart problems and influences the utilization of health care resources [27].

^f^The wording of the corresponding question is: “For the past six months at least, to what extent have you been limited because of a health problem in activities people usually do?” Possible answers: Severely limited, limited but not severely, not limited. Our binary dependent variable is set zero if no limitation is indicated and one otherwise. Both mental and physical health issues do have an impact on individuals’ activities. This is supported by the correlation matrix between our dependent variable and the various mental and physical conditions. For both types of conditions the correlation with the dependent variable is within a range of 0.06 and 0.29.

^g^For a general discussion of aspects of heterogeneity in ordered choices and a detailed description of the generalized ordered probit model see Greene and Henscher [28].

^h^The order condition in the generalized ordered probit model requires that the predicted probabilities are in the (0; 1) interval.

^i^The related user-written Stata program regoprob2 is available at the SSC archive.

^j^In our sample, over 23 % of those aged above 65 years have more than 11 visits while this applies to only 12 % for those 65 or younger.

^k^As additional country fixed effect the GDP per capita (ppp) was included. However, no matter which combination of variables was jointly estimated, the model did not converge.

^l^The full estimation results of the three alternative specifications are available upon request.

## Competing interests

The authors declare that they have no competing interests.

## Acknowledgments

For helpful comments we would like to thank Peter Zweifel, the participants of the iHEA World Congress 2011 in Toronto, of the German Health Economic Association annual meeting 2011 in Bayreuth and of the 32nd Nordic Health Economists’ Study Group (NHESG) meeting in Odense.

## Authors’ contribution

CP, AS and US have worked collaboratively during all stages of the project. All authors read and approved the final manuscript.
